# Repetitive nerve stimulation on survival in amyotrophic lateral sclerosis

**DOI:** 10.3389/fneur.2023.1244385

**Published:** 2023-08-16

**Authors:** Yahui Zhu, Jiongming Bai, Mao Li, Hongfen Wang, Jiao Wang, Xusheng Huang

**Affiliations:** ^1^Medical School of Chinese PLA, Beijing, China; ^2^Department of Neurology, The First Medical Center, Chinese PLA General Hospital, Beijing, China; ^3^College of Medicine, Nankai University, Tianjin, China

**Keywords:** repetitive nerve stimulation, amyotrophic lateral sclerosis, survival, marker, ALS functional rating scale-revised score

## Abstract

**Objective:**

No previous studies investigated the association between decrement of low-frequency repetitive nerve stimulation (LF-RNS) and amyotrophic lateral sclerosis (ALS) survival. We aim to study the relationship between decrement and survival in ALS.

**Methods:**

Sporadic ALS patients diagnosed at the Department of Neurology, the First Medical Center, Chinese PLA General Hospital from January 2018 to December 2019 were enrolled in this study. Experienced neurologists followed up the participants regularly every 6 months until January 2022. A decremental response of 10% or greater at least in one muscle was considered positive. According to the decrement, the participants were divided into LF-RNS (+) and LF-RNS (−) groups.

**Results:**

One hundred and eighty-one sporadic ALS patients were recruited in our study, including 100 males and 81 females. Among them, 10 cases (5.5%) were lost to follow-up, 99 cases (54.7%) died, and 72 patients (39.8%) were still alive at the last follow-up. The median survival time of all ALS patients in this study was 42.0 months. There was no significant difference of median survival in LF-RNS(+) group and LF-RNS(−) group (*p* = 0.159, Kaplan–Meier method). In multivariate Cox regression analysis, age of onset, diagnostic delay, and ALS Functional Rating Scale-Revised (ALSFRS-R) score were associated with ALS survival, but the decrement was not correlated with ALS survival (*p* = 0.238).

**Conclusion:**

The decrement in accessory and ulnar nerves was not associated with the survival of ALS. The decrement of LF-RNS could not be an electrophysiological marker to predict ALS survival.

## 1. Introduction

Amyotrophic lateral sclerosis (ALS) is a progressive neurodegenerative disease, characterized by dysarthria, dysphagia, limb weakness, muscle atrophy and respiratory failure. ALS is caused by degeneration of upper and lower motor neurons, but the etiology is not yet clear (1). There is no effective cure for ALS so far. The general survival time of ALS is 3–5 years from symptom onset to death (2). Increasing studies have been focused on markers that predict survival in ALS, for example, ALS Functional Rating Scale-Revised (ALSFRS-R) score, respiratory function, age at disease onset, diagnostic delay and different onset forms (3–5).

Repetitive nerve stimulation (RNS) is a technique widely used to assess the neuromuscular junction function. In 1959, Mulder et al. (6) first described a decremental response to low-frequency RNS (LF-RNS) in ALS patients. Subsequently, other studies have also evaluated the abnormality of LF-RNS in patients with ALS. We previously studied the features of LF-RNS in Chinese patients with ALS. Our previous study has demonstrated that the decremental response to LF-RNS (≥10%) in at least one muscle was detected in 56.8% of the patients and was most commonly seen in trapezius and deltoid (7). A positive RNS usually indicates the abnormality of the neuromuscular junction (NMJ). Some studies have suggested that ALS is characterized by progressive denervation and chronic reinnervation, and the immature structure of new NMJ leads to neurotransmitter delivery disorders and thus abnormal RNS (8). This is also termed the “dying forward” theory. Other studies have suggested that the abnormal RNS in ALS was due to the fact that MN pathology begins with the NMJs and distal axons, and then proceeds in reverse, as a “dying back” pattern (7, 9).

In addition, the relationship between decrement and disease progression in ALS has been studied. Wang et al. (10) found that the positive decremental responses to LF-RNS in ALS indicated faster disease progression and helped to assess prognosis. In one study, a greater decrement of RNS was observed in a group of patients with rapidly progressive ALS than patients with slowly progressive ALS (11). However, other studies have shown no statistical difference in progression rates between RNS(+) and RNS(−) groups (7, 12, 13). Previous studies have shown that progression rate is highly correlated with its survival time (14). Currently, there are no studies on the correlation between decrement and ALS survival.

Due to the high positive rate of LF-RNS in ALS patients, and the availability and repeatability of the procedure, it is worth exploring whether it can be used to predict the survival of ALS. Therefore, the purpose of this study was to explore the association between decrement and ALS survival.

## 2. Materials and methods

According to El Escorial revised criteria (15), consecutively sporadic ALS patients (including clinically definite or clinically probable ALS patients) diagnosed at the Department of Neurology, the First Medical Center, Chinese PLA General Hospital from January 2018 to December 2019 were enrolled in this study. The exclusion criteria were as follows: (1) patients with a history of poliomyelitis, (2) patients with spinal cord tumor, and (3) patients with other diseases affecting peripheral nerves, neuromuscular junction (NMJ), or muscles. Experienced neurologists followed up the participants regularly every 6 months with telephone or face-to-face interview until January 2022.

Muscle strength was assessed using modified Medical Research Council (MRC) scale. The electrophysiological studies were performed in a keypoint workstation machine (31A06, Alpine Biomed ApS, Denmark), including RNS, electromyography (EMG) and nerve conduction studies. The measured limb was in a relaxed state. The skin temperature of the examined muscle was maintained at 32°C or above throughout the examination. On the side with more severe symptom involvement, LF-RNS was performed. For asymptomatic patients on both upper limbs, RNS examination was performed on the right side. LF-RNS was performed in trapezius for the accessory nerve and abductor digiti minimi (ADM) for the ulnar nerve in all ALS patients. The accessory nerve was stimulated on the posterior border of the sternocleidomastoid and the ulnar nerve was stimulated on the wrist. The surface recording electrodes were used to record over the belly of the trapezius and the ADM. The reference electrodes were placed on the tendon of the tested muscles. Compound muscle action potential (CMAP) was recorded in the trapezius and ADM of 10 stimuli at a low frequency of 3 Hz in the accessory nerve and ulnar nerve, separately. A decrement of the peak-to-peak amplitudes of the CMAP of the 4th–1st responses was measured. A decremental response of 10% or greater at least in one muscle was considered positive, as recommended by suggestions of the AAEM Quality Assurance Committee (16). None of our ALS patients received any cholinesterase inhibitors or other drugs that may affect neuromuscular junction function before the assessment of RNS.

Baseline clinical data of ALS patients, including age at disease onset, diagnostic delay, site of onset (bulbar versus spinal), body mass index (BMI), ALSFRS-R score and disease progression rates, were obtained. Diagnostic delay was defined as the interval (in months) between symptom onset and diagnosis. Disease severity was assessed using the ALSFRS-R scale. Disease progression rates were calculated by the following formula: ΔFS = (48-ALSFRS-R score at time of diagnosis)/Duration from symptom onset to diagnosis (months).

For patients who died, the survival time was from disease onset to death, tracheostomy, or censoring date (January 31, 2022). For patients who lost to follow-up, survival time was calculated from onset of disease to the last contact. All patients were followed up by neurologists every 6 months. If the neurologists failed to contact patients by phone twice, patients were considered to be lost at follow-up. This study was approved by the Ethics Committee of Chinese PLA General Hospital and written informed consent was obtained from participants.

All statistical analyses were performed using SPSS 22.0. Based on the decrement, participants were classified into LF-RNS(+) and LF-RNS(−) groups. Comparison of continuous variables between groups were calculated with Student’s *t*-tests or Mann–Whitney U-tests. Enumeration data between groups were compared using Chi-squared test. Survival comparison between groups were performed with Kaplan–Meier curves and log-rank tests. Continuous variables were classified into appropriate forms to fit proportional hazards. Univariate and multivariate Cox regression analyses were performed to determine the variables associated with ALS survival. Data for continuous variables were expressed as mean ± standard deviation (SD) or median with quartile. The significance level was set at *p* < 0.05.

## 3. Results

Totally, 181 sporadic ALS patients were recruited in our study, including 100 males and 81 females. Among them, 10 cases (5.5%) were lost to follow-up, 99 cases (54.7%) died, and 72 patients (39.8%) were still alive at the last follow-up. No patient underwent tracheostomy in our study. The epidemiological characteristics of all ALS patients were shown in Table 1. Abnormal decrement in trapezius and ADM were found in 46.4 and 8.8% of the muscles, respectively. The mean CMAP decremental percentage of accessory and ulnar nerves were 10.8 ± 7.9 and 4.6% ± 4.7%. Then, we further analyzed the relationship between CMAP amplitude and decrement, and the results showed that CMAP amplitude was negatively correlated with decrement (*r* = −0.301, *p* < 0.001) in ulnar nerve. According to the decrement, ALS patients were divided into two groups. No significant differences were shown in gender, site of onset, age of onset, diagnostic delay, BMI, ALSFRS-R score and disease progression rates between LF-RNS(+) and LF-RNS(−) groups (Table 1).

**Table 1:** Clinical features of ALS patients based on the LF-RNS results.

	All patients	LF-RNS(+)	LF-RNS(−)	*p*
Number	181	86	95	
Gender (male/female)	100/81	47/39	53/42	0.878
Site of onset (Bulbar/Limb)	40/141	18/68	22/73	0.718
Age of onset (Years)	52.0 ± 10.7	51.7 ± 11.0	52.2 ± 10.5	0.728
BMI	23.5 ± 3.2	23.2 ± 3.4	23.7 ± 3.0	0.271
Diagnostic delay (months)	11.0 (6.0, 19.0)	11.0 (7.0, 19.0)	12.0 (6.0, 19.0)	0.821
ALSFRS-R	38.7 ± 5.9	38.4 ± 6.4	38.9 ± 5.4	0.595
Disease progression rates (score/month)	0.55 (0.33, 1.00)	0.55 (0.33, 1.02)	0.56 (0.31, 1.00)	0.794
Muscle strength of trapezius (normal/unnormal)	164/17	77/9	87/8	0.638
Muscle strength of ADM (normal/unnormal)	96/85	58/28	38/57	2.21 × 10^−4^

LF-RNS, low frequency repetitive nerve stimulation; BMI, body mass index; ALSFRS-R, ALS functional rating scale-revised; ADM, abductor digiti minimi. Age of onset, BMI, and ALSFRS-R were expressed as mean ± standard deviation (SD), and diagnostic delay and disease progression rates were expressed as median with interquartile range.

**Figure 1:**
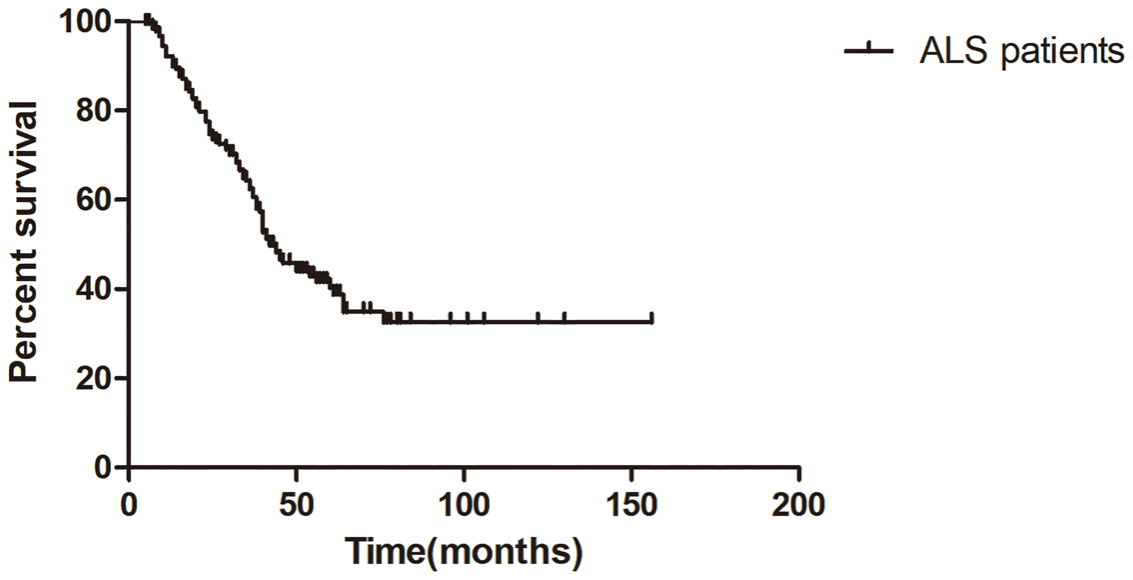
Kaplan–Meier survival plot in all ALS patients from symptom onset.

**Figure 2:**
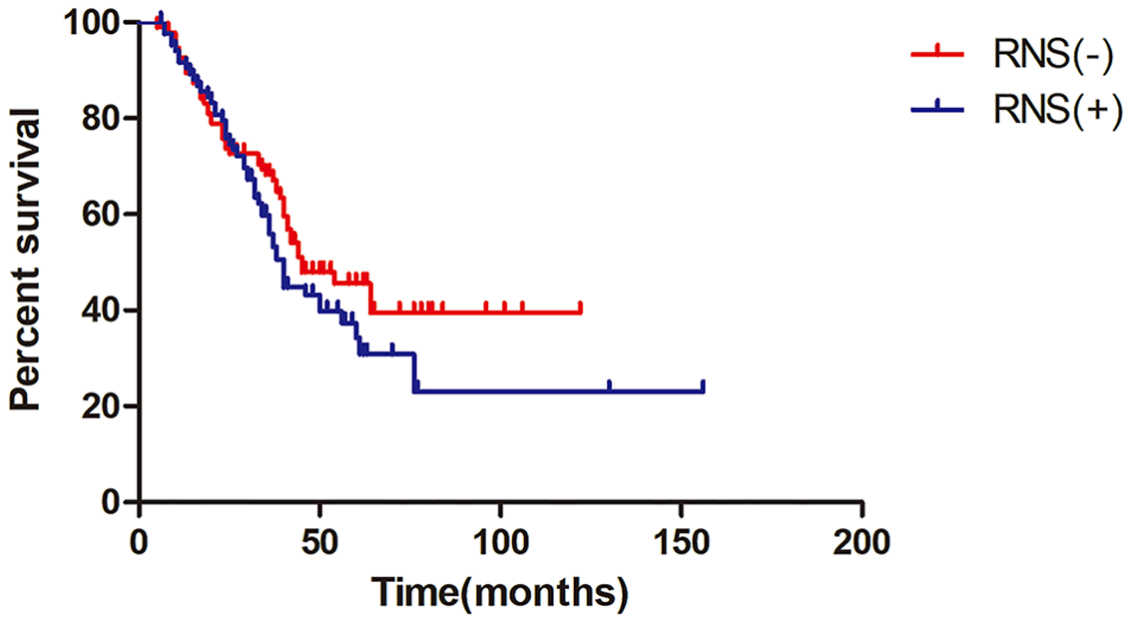
Kaplan–Meier survival plot in ALS patients with LF-RNS(+) versus with LF-RNS(−). LF-RNS, low frequency repetitive nerve stimulation.

The median survival time of all ALS patients in this study was 42.0 months (95%CI = 35.7–48.3), as shown in Kaplan–Meier analysis (Figure 1). In addition, patients with LF-RNS(+) had shorter median survival than those with LF-RNS(−) (40.0 months vs. 45.0 months), but there was no significant difference (*p* = 0.159) (Figure 2).

In univariate Cox regression analysis (Table 2), several parameters were identified to predict longer survival in ALS patients, including limb onset, low age of onset, long diagnostic delay, and high ALSFRS-R score. However, the decrement was not significantly associated with the survival of ALS. Similarly, in multivariate Cox regression analysis, results showed that low age of onset, long diagnostic delay, and high ALSFRS-R score were significantly associated with longer survival time in ALS patients. Furthermore, the decrement was still not correlated with ALS survival.

**Table 2:** Univariate and multivariate Cox survival analysis results in ALS patients.

		Univariate analysis			Multivariate analysis		
Variables		HR	95%CI	*p*	HR	95%CI	*p*
Onset form	Limb vs. Bulbar	0.604	0.390–0.936	0.024	0.747	0.458–1.218	0.242
Age of onset	Higher age of onset vs. lower age of onset than 52	2.150	1.400–3.304	4.75 × 10^−4^	2.119	1.353–3.320	0.001
BMI	Higher BMI vs. lower BMI than 23	0.799	0.536–1.189	0.269	0.872	0.561–1.358	0.545
Diagnostic delay	Longer diagnostic delay vs. shorter diagnostic delay than 11 months	0.420	0.279–0.631	2.90 × 10^−5^	0.292	0.187–0.457	<0.001
ALSFRS-R	Higher ALSFRS-R vs. lower ALSFRS-R than 38	0.553	0.371–0.825	0.004	0.481	0.310–0.745	0.001
LF-RNS	LF-RNS(+) vs. LF-RNS(−)	1.324	0.892–1.967	0.164	1.272	0.853–1.896	0.238
Gender	Female vs. male	0.844	0.567–1.256	0.403			

BMI, body mass index; ALSFRS-R, ALS functional rating scale-revised; LF-RNS, low frequency repetitive nerve stimulation; HR, hazard ratio; CI, confidence interval.

## 4. Discussion

To our knowledge, our study is the first large-scale study to explore the association between decrement and ALS survival. An association between decrement in accessory and ulnar nerves and ALS survival was not observed. We found that decrement could not predict the survival of ALS.

Previous studies have shown that the disease progression rate in the LF-RNS(+) group was faster than that in the LF-RNS(−) group. Therefore, they considered positive decrements in RNS were an indicator of disease progression and active period (10). However, Alanazy et al. (17) found no significant association between decrement in RNS and handgrip fatigue, slow vital capacity (SVC), or more rapid worsening of SVC over time. The results showed no correlation between abnormal decrement in RNS and clinical measurements, suggesting that RNS may not be useful for monitoring ALS progression. In the study of Hu et al., the CMAP amplitude was linearly correlated with decremental percentage in accessory and axillary nerve in ALS patients. This further supports that the decremental response in RNS in ALS patients can be attributed to immature sprouts and unstable NMJ conduction by degenerated axons. In addition, their study showed no significant difference in progression rates between the RNS(+) and RNS(−) groups. The study showed that RNS(+) only reflected underlying neurodegeneration and could not be used to monitor disease progression (12). Our previous study and this study also showed that there was no significant difference in the rate of disease progression between the RNS(+) and RNS(−) groups (7).

In general, faster disease progression is associated with shorter survival. Previous studies and this study have found no significant difference in the rate of disease progression between the RNS (+) and RNS (−) groups, which also supported our findings to some extent. However, disease progression in ALS patients is not entirely linear and it may progress faster or slower at some stage. Therefore, the rate of disease progression does not fully predict the survival time of ALS. While previous studies have focused on decrement and the rate of disease progression in ALS, our study directly assesses the association between decrement and ALS survival time. Compared with previous studies, this study can more accurately show whether decrement can assess ALS prognosis.

Clinical parameters such as age of onset, diagnostic delay, and ALSFRS-R score can be used to predict ALS survival (18). Our study also found that age of onset, diagnostic delay, and ALSFRS-R score were associated with survival time, which was consistent with previous studies.

There are some limitations in our study. First, although we followed up the outcomes of ALS patients and used a Cox model to analyze the relationship between decrement and survival, we did not measure the change of decrement during follow up. Second, RNS decrement abnormalities may occur in bulbar district of ALS patients. However, decrement of bulbar district was not studied. Third, RNS decrement of median nerve was not examined in this study.

In conclusion, our study did not find any association between decrement of LF-RNS in accessory and ulnar nerves and ALS survival. Although LF-RNS is a well-tolerated and reproducible test, it can not be used as an electrophysiological marker for predicting survival in ALS.

## Data availability statement

The raw data supporting the conclusions of this article will be made available by the authors, without undue reservation.

## Ethics statement

The studies involving humans were approved by Ethics Committee of Chinese PLA General Hospital. The studies were conducted in accordance with the local legislation and institutional requirements. The participants provided their written informed consent to participate in this study.

## Author contributions

YZ, JB, ML, HW, JW, and XH contributed to the study conception and design. Material preparation, data collection, and analysis were performed by YZ, JB, and ML. The first draft of the manuscript was written by YZ and JB. YZ, JB, ML, HW, JW, and XH commented on previous versions of the manuscript. All authors contributed to the article and approved the submitted version.

## Conflict of interest

The authors declare that the research was conducted in the absence of any commercial or financial relationships that could be construed as a potential conflict of interest.

## Publisher’s note

All claims expressed in this article are solely those of the authors and do not necessarily represent those of their affiliated organizations, or those of the publisher, the editors and the reviewers. Any product that may be evaluated in this article, or claim that may be made by its manufacturer, is not guaranteed or endorsed by the publisher.

## References

[1] Logroscino G Traynor BJ Hardiman O Chio A Couratier P Mitchell JD. Descriptive epidemiology of amyotrophic lateral sclerosis: new evidence and unsolved issues. J Neurol Neurosurg Psychiatry. (2008) 79:6–11. doi: 10.1136/jnnp.2006.104828, PMID: 18079297

[2] Chio A Logroscino G Hardiman O Swingler R Mitchell D Beghi E. Prognostic factors in ALS: a critical review. Amyotroph Lateral Scler. (2009) 10:310–23. doi: 10.3109/17482960802566824, PMID: 19922118 PMC3515205

[3] Chen L Zhang B Chen R Tang L Liu R Yang Y. Natural history and clinical features of sporadic amyotrophic lateral sclerosis in China. J Neurol Neurosurg Psychiatry. (2015) 86:1075–81. doi: 10.1136/jnnp-2015-310471, PMID: 26124198

[4] Kaufmann P Levy G Thompson JL Delbene ML Battista V Gordon PH. The ALSFRSr predicts survival time in an ALS clinic population. Neurology. (2005) 64:38–43. doi: 10.1212/01.WNL.0000148648.38313.64 15642901

[5] Wei Q Chen X Zheng Z Guo X Huang R Cao B. The predictors of survival in Chinese amyotrophic lateral sclerosis patients. Amyotroph Lateral Scler Frontotemporal Degener. (2015) 16:237–44. doi: 10.3109/21678421.2014.993650, PMID: 25581512

[6] Mulder DW Lambert EH Eaton LM. Myasthenic syndrome in patients with amyotrophic lateral sclerosis. Neurology. (1959) 9:627–31. doi: 10.1212/WNL.9.10.627 14425103

[7] Sun X Liu W Chen Z Ling L Yang F Wang H. Repetitive nerve stimulation in amyotrophic lateral sclerosis. Chin Med J. (2018) 131:2146–51. doi: 10.4103/0366-6999.240798, PMID: 30203787 PMC6144859

[8] Kiernan MC Vucic S Cheah BC Turner MR Eisen A Hardiman O. Amyotrophic lateral sclerosis. Lancet. (2011) 377:942–55. doi: 10.1016/S0140-6736(10)61156-7 21296405

[9] Fischer LR Culver DG Tennant P Davis AA Wang M Castellano-Sanchez A. Amyotrophic lateral sclerosis is a distal axonopathy: evidence in mice and man. Exp Neurol. (2004) 185:232–40. doi: 10.1016/j.expneurol.2003.10.004, PMID: 14736504

[10] Wang Y Xiao Z Chu H Liang J Wu X Dong H. Correlations between slow-rate repetitive nerve stimulation and characteristics associated with amyotrophic lateral sclerosis in Chinese patients. J Phys Ther Sci. (2017) 29:737–43. doi: 10.1589/jpts.29.737, PMID: 28533621 PMC5430284

[11] Bernstein LP Antel JP. Motor neuron disease: decremental responses to repetitive nerve stimulation. Neurology. (1981) 31:202–7. doi: 10.1212/WNL.31.2.202 6258105

[12] Hu F Jin J Kang L Jia R Qin X Liu X. Decremental responses to repetitive nerve stimulation in amyotrophic lateral sclerosis. Eur Neurol. (2018) 80:151–6. doi: 10.1159/000494670, PMID: 30463071

[13] Shang L Chu H Lu Z. Can the large-scale decrement in repetitive nerve stimulation be used as an exclusion criterion for amyotrophic lateral sclerosis? Front Neurol. (2020) 11:101. doi: 10.3389/fneur.2020.00101, PMID: 32184752 PMC7059024

[14] Westeneng HJ Debray TPA Visser AE van Eijk RPA Rooney JPK Calvo A. Prognosis for patients with amyotrophic lateral sclerosis: development and validation of a personalised prediction model. Lancet Neurol. (2018) 17:423–33. doi: 10.1016/S1474-4422(18)30089-9, PMID: 29598923

[15] Brooks BR Miller RG Swash M Munsat TL. World Federation of Neurology Research Group on motor neuron diseases. El Escorial revisited: revised criteria for the diagnosis of amyotrophic lateral sclerosis. Amyotroph Lateral Scler Other Motor Neuron Disord. (2000) 1:293–9. doi: 10.1080/146608200300079536 11464847

[16] AAEM Quality Assurance Committee. American Association of Electrodiagnostic Medicine. Literature review of the usefulness of repetitive nerve stimulation and single fiber EMG in the electrodiagnostic evaluation of patients with suspected myasthenia gravis or Lambert-Eaton myasthenic syndrome. Muscle Nerve. (2001) 24:1239–47. doi: 10.1002/mus.1140 11494281

[17] Alanazy MH Hegedus J White C Korngut L. Decremental responses in patients with motor neuron disease. Brain Behav. (2017) 7:e00846. doi: 10.1002/brb3.846, PMID: 29201547 PMC5698864

[18] Cao B Wei Q Ou R Zhang L Hou Y Chen Y. Neurophysiological index is associated with the survival of patients with amyotrophic lateral sclerosis. Clin Neurophysiol. (2019) 130:1730–3. doi: 10.1016/j.clinph.2019.05.012, PMID: 31164255

